# Female Fertility Affects Men's Linguistic Choices

**DOI:** 10.1371/journal.pone.0027971

**Published:** 2012-02-08

**Authors:** Jacqueline M. Coyle, Michael P. Kaschak

**Affiliations:** 1 Human Factors and Systems Department, Embry-Riddle Aeronautical University, Daytona Beach, Florida, United States of America; 2 Psychology Department, Florida State University, Tallahassee, Florida, United States of America; Catholic University of Sacro Cuore, Italy

## Abstract

We examined the influence of female fertility on the likelihood of male participants aligning their choice of syntactic construction with those of female confederates. Men interacted with women throughout their menstrual cycle. On critical trials during the interaction, the confederate described a picture to the participant using particular syntactic constructions. Immediately thereafter, the participant described to the confederate a picture that could be described using either the same construction that was used by the confederate or an alternative form of the construction. Our data show that the likelihood of men choosing the same syntactic structure as the women was inversely related to the women's level of fertility: higher levels of fertility were associated with lower levels of linguistic matching. A follow-up study revealed that female participants do not show this same change in linguistic behavior as a function of changes in their conversation partner's fertility. We interpret these findings in the context of recent data suggesting that non-conforming behavior may be a means of men displaying their fitness as a mate to women.

## Introduction

Speaking is about making choices. What do I want to say? What words should I use? Should I speak in the active voice, or should the passive voice be used? The choices that we make when we speak are largely unconscious [1], [2] and are driven by numerous considerations. As an example, the selection of some language patterns over others helps to establish one's identity and group membership [3]–[5]. Furthermore, the use of particular language patterns can reflect both a person's disposition toward their conversational partner, and the language use of their conversational partner itself. It is well documented that conversational partners align their behavior on all levels of linguistic structure, such as rate of speech [6], lexical choices [7], or use of particular syntactic constructions [8]. Linguistic alignment is often interpreted as a sign of affiliation between speakers. Giles et al. [6] have argued that speakers align their linguistic behavior to signal affiliation with a conversational partner, and diverge from the linguistic behavior of their conversational partner as a means of creating social distance. This argument fits within a larger framework of research showing that alignment and mimicry of behavior facilitates interpersonal communication, and leads to increased liking between the parties to the interaction [9], [10].

### Linguistic Alignment and Mating Goals

Alignment of linguistic and non-linguistic behavior (or lack thereof) is a key and meaningful component of social interaction [2], [11], [9], and it has been demonstrated that the degree of alignment between the parties involved in an interaction predicts a variety of outcomes. Of particular relevance to the present study, Ireland et al. [12] found that the degree of matching in language style between conversational partners was positively correlated both with the likelihood that a romantic relationship between the partners would be initiated, and with the likelihood that a relationship was stable. Though the correlational nature of this study does not permit a strong conclusion about whether linguistic matching is a cause or byproduct of affiliation within the developing relationships, the finding suggests that alignment of linguistic behavior with a potential romantic partner may be an important aspect of signaling one's interest in that partner, and initiating and maintaining a relationship.

Although it is intuitive that alignment serves as a means of building affiliation between potential romantic partners, the role of linguistic alignment in attracting a potential mate may not be so straightforward. Creativity is an attractive quality in mates [13]–[15], and evidence suggests that priming males with mating goals leads to displays of creativity [16], increases in non-conforming behavior [17], and risk taking [18]–[20]. Such behavioral displays have been interpreted as demonstrations of one's fitness as a mate. Rosenberg and Tunney [21] report this sort of display in the context of language use. In their study, males primed with mating goals tended to use lower frequency words (signaling creativity and depth of vocabulary) compared to males primed with a friendship motivation. These findings suggest the possibility that there are circumstances under which men may not align their linguistic behavior with a female conversation partner as a means of attracting her as a potential mate.

The study reported below was designed to assess whether men's linguistic alignment with a female partner would be affected by exposure to cues to the woman's fertility level. Male participants were asked to perform a picture description task with a female confederate. Men interacted with the female confederates who were at various points in their menstrual cycle at the time of the interaction. Miller and Maner [20], [22], [23] demonstrate that men are sensitive to subtle cues to female fertility (e.g., changes in facial skin tone, vocal pitch, and scent), and that detection of fertility cues activates mating goals. Men find women displaying cues to high fertility more attractive and desirable than women not displaying these cues [22], and detection of cues to fertility leads to increases in men's testosterone levels [23].

We assess linguistic alignment via a phenomenon known as *structural priming*, or the tendency to repeat syntactic constructions across utterances [24]. For example, after having produced (or heard) a token of the double object construction (DO: *Meghan gave Michael a toy*), a person is more likely to subsequently produce another DO construction (*The captain sent the first mate a message*) than to produce the alternative prepositional object construction (PO: *The captain sent a message to the first mate*). The DO and PO constructions have essentially the same meaning, and thus the choice between constructions is a syntactic rather than semantic choice. Structural priming is a robust phenomenon (see [25]), and it occurs largely outside of a speaker's awareness [1]. Indeed, structural priming may well reflect implicit learning within the language production system [26], [27]. On critical trials in our study, the confederate described a picture to the participant using either the DO or PO construction. Immediately thereafter, the participant had the opportunity to describe a picture depicting someone transferring an object to someone else (i.e., a picture eliciting the production of the DO and PO constructions). The question is whether the participants' description employs the same construction as the confederate employed on the preceding utterance.

We considered two possibilities for how exposure to fertility cues would affect structural priming. Previous work has shown that men find fertile women to be more attractive [22] and that individuals respond to attractive potential mates by aligning their behavior with that of their partner [12], [20]. This literature, in conjunction with the broader literature showing a relationship between behavioral and linguistic alignment and liking [9], [6], suggests that men who are exposed to fertility cues may show an increase in linguistic alignment with their female partners. At the same time, other reports suggest that men may respond to attractive women, or to situations in which they are thinking about mating goals and relationships, by producing non-conforming or creative behavior [17]. Rosenberg and Tunney's [21] results demonstrate that such considerations may extend to language use. These data suggest that men may not align their linguistic behavior with fertile women as a means of displaying their fitness as a mate. Thus, increases in fertility will lead to a decrease in linguistic alignment.

## Experiment 1

### Methods

#### Ethics Statement

This project was approved by the IRB at Florida State University on 07/15/2008. Written consent was obtained from each participant.

#### Participants

The participants were 123 male undergraduates from Florida State University. Due to our research goals, two participants who self-reported a homosexual orientation were excluded from the data analysis.

#### Confederates and Experimenters

Five undergraduate women not taking hormonal contraceptives were confederates. They served as confederates throughout their menstrual cycles. Menstrual cycles were tracked by having confederates report the onset and end of each menses to JMC. Our training and handling of the confederates followed the procedures outlined by Miller and Maner ([20], Study 3). To avoid issues associated with having another male present during the experiment, all experimenters were females taking hormonal birth control (and thus not presenting cues to fertility).

#### Materials

**Figure 1:**
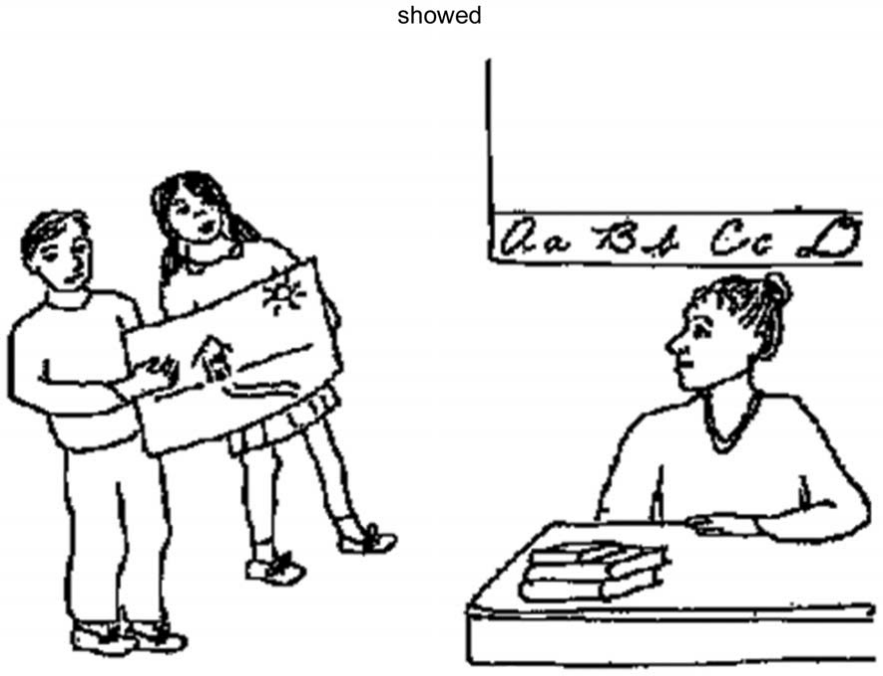
Example picture from the picture description task.

Two sets of pictures (“description sets”; one for the confederate, and one for the participant) were constructed. Each consisted of 17 pictures: 8 critical pictures that could be described using the DO or PO construction, and 9 filler pictures. Each picture had a verb typed above it, which was to be used in generating a sentence to describe the picture (see Figure 1). The critical pictures used by the confederate were scripted to be described with either a DO or PO construction (4 DO descriptions and 4 PO descriptions). Confederates produced the same set of picture descriptions for every participant. The pictures in both sets were put into a fixed order, such that each critical picture described by the confederate was immediately followed by a critical picture for which the participant could use either the DO or PO construction to generate a description. Critical pictures used the same verb as the confederate's picture half of the time, and used a different verb half of the time^1^. The manipulation of construction type (DO vs. PO) and verb repetition (same verb vs. different verb) was intended to provide variability across trials. As we did not counterbalance critical pictures across construction type or verb repetition, meaningful conclusions about the effects of these variables cannot be drawn. Because of this, and because the effects of construction type and verb repetition are orthogonal to the effects of conception risk (as the same items are given to every participant), these variables are not included in the analysis reported below. A duplicate of each description set was created to be used for identifying the picture one's partner just had described (the “matching sets”). Matching sets were shuffled before each use.

#### Questionnaires

Participants were given a questionnaire packet to assess demographic information (e.g., age and sex), their sexual orientation and relationship status (in a committed relationship or not), and their impressions of the confederate (from 1 = “not at all” to 5 = “extremely”) on several dimensions: intelligent, flirtatious, outgoing, attractive, happy, sad, angry, and sexually aroused. We also included a behavioral measure of conformity, as one's tendency toward conformity might affect the degree of linguistic alignment displayed toward a conversational partner. The measure was a slightly modified version of the conformity measure used by Griskevicius et al. [17]. It consisted of 6 subjective choices requiring participants to indicate their preference between two similar items (e.g., Mercedes-Benz vs. BMW). Participants were told that the form had already been filled out by two other participants, and on critical responses the prior responses seen by the participant were scripted so that there was an apparent consensus (i.e., the other participants chose the same item). Conformity is measured by calculating the proportion of conforming responses on these critical items.

#### Procedure

To begin each session, the participant and confederate were seated at a small table to fill out consent forms. The participant and confederate were put in close proximity to expose the participant to the fertility cues (skin tone, scent, etc.) displayed by the confederate. The confederate and participant were free to interact during this time period. As in Miller and Maner [20], the confederates were told to keep conversation to a minimum, to remain expressively neutral during the interactions, and to appear polite but not overly interested in the participant. Given that a relatively brief exposure to fertility cues (e.g., briefly smelling a T-shirt worn by a female experimenter at a given point during her menstrual cycle, or spending a few minutes interacting with a female confederate during a group task [20]) appears to be all that is needed to activate mating goals, it was our sense that this initial period in our study would be sufficient to produce a similar activation of goals. After a couple minutes, the participant and confederate were seated at different tables separated by a divider. The participant and confederate then engaged in the picture description task developed by Branigan et al. [8]. The participant and confederate were both given two stacks of pictures: a description stack, and a matching stack. They were told that they would take turns describing pictures to one another. The descriptions were to be one sentence, and were to use the verb on top of the picture. When a picture was described, the listener was to find the matching picture in his/her matching stack. They would then describe the next picture in their description stack. The task would continue until all pictures had been described and matched. The task proceeded as described, and the confederate always went first. After finishing the task, the participant completed the questionnaires described above.

#### Design and Analysis

The IV of primary interest was the fertility level of the confederates. Following previous research [28], [20], fertility level was operationalized as *conception risk*, with risk values (from [29]) being estimated according to the day of the confederate's menstrual cycle on which the interaction took place. Conception risk values range from 0 to .10 in our data. Higher risk values indicate a higher level of fertility.

The picture description task was audio recorded. The participants' responses to the critical pictures were scored as DO, PO, or “other” following the criteria described in Kaschak [30]. Trials on which an “other” response was made (4% of trials) were excluded from subsequent analyses. The dependent measure for our analyses was Match, which coded whether participants used the same syntactic construction to describe the target picture as the confederate used in producing the prime sentence (matches were coded as 1, and mismatches were coded as 0).

**Figure 2:**
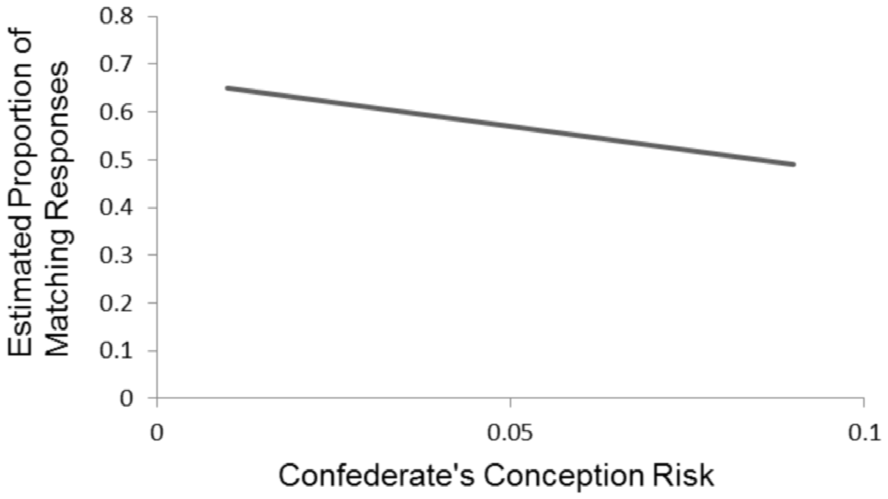
Estimated proportion of trials on which participants matched the syntactic construction of the confederate as a function of confederates' conception risk.

Mixed logit analysis of the target descriptions was performed to predict the logit-transformed likelihood of a target using the same syntactic construction as the confederate's prime sentence. We performed an initial analysis with Match as the dependent measure, and participants and items as crossed random factors. The following variables were considered as potential predictors: conception risk, relationship status, the interaction of risk and relationship status, conformity, the interaction of conformity and conception risk, and the 8 variables on which the participant rated the confederate. To avoid issues with collinearity, all variables were grand-mean centered before being entered into the analysis. We began with a model that included conception risk as the only predictor. To yield more interpretable coefficients for conception risk, we multiplied the risk values by 100 to convert them to a percentage (ranging from 0 to 10) for the analysis; note that in Figure 2 conception risk has been converted back to a proportion. Conception risk was a significant predictor of matching. We then assessed whether the addition of any of the other predictors improved model fit. Only participants' ratings of the confederate's flirtatiousness improved model fit. Our final model also included random slopes across items for both predictors. The regression analyses were performed using the lme4 package of R [31].

### Results and Discussion

#### Preliminary Analyses

We began by analyzing participants' ratings of the confederates to ensure that changes in conception risk were not accompanied by other changes that might account for the effects of risk on structural priming. As in Miller and Maner's [20] Experiment 3 (on which this study was modeled), regression analysis in which participants' ratings were used to predict conception risk revealed that none of the rating variables were significant predictors of risk (all *p*'s>.11), and that the combination of all the variables did not account for a significant amount of variability in conception risk [*F*<1]^2^. The finding that participants' ratings of the confederates' attractiveness did not change as a function of conception risk might appear to be at odds with other findings suggesting that men find fertile women to be more attractive [32]–[35]. However, in those studies it is likely that changes in style of dress, use of makeup, and other factors (e.g., flirting) accounted for the change in ratings of attractiveness. In studies such as ours, where style of dress, use of makeup, and the like are controlled across the menstrual cycle, changes in fertility do not appear to be associated with changes in perceived attractiveness [20].

The second preliminary analysis we performed was intended to confirm that our method produced a reliable structural priming effect. Our dependent variable, Match, assesses the odds of the participant matching the construction that was just produced by the confederate. Structural priming is demonstrated when participants match the construction produced by the confederate on more than 50% of the trials. We computed the proportion of trials on which each participant matched the construction produced by the confederate, and conducted a one-sample t-test comparing this mean to a proportion of .5 (i.e., no demonstrated structural priming). The mean proportion of matching trials (*M* = .58, *SD* = .14) was statistically different from .5 [*t*(120) = 6.11, *p*<.001], demonstrating that our study produced the standard structural priming effect.

#### Main Analysis

**Figure 3:**
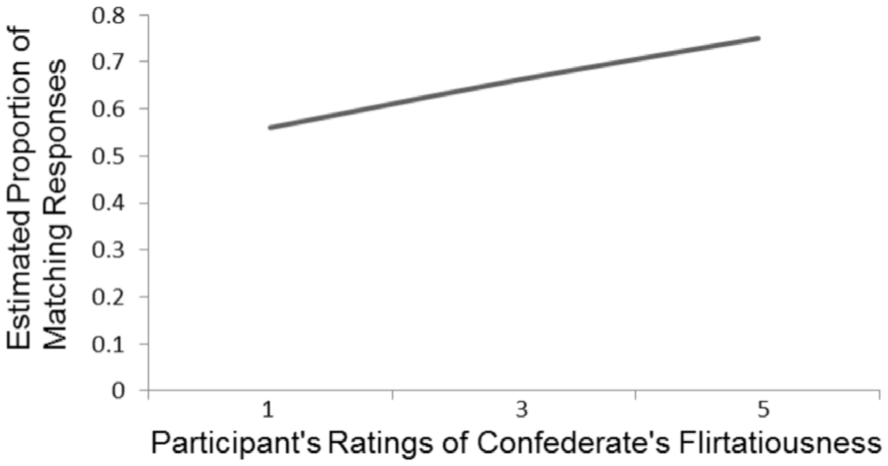
Estimated proportion of trials on which participants matched the syntactic construction of the confederate as a function of the participants' perception of the confederates' flirtatiousness.

The mixed logit regression model predicting the log odds of the participant matching the syntactic construction produced by the confederate on the immediately preceding trial is presented in Table 1. The critical result of our experiment is the finding that conception risk affects the odds of participants matching the syntactic constructions produced by the confederate (*p* = .003). As conception risk increases, the odds of participants matching the constructions produced by the confederate decrease (see Figure 2). We explored this finding by examining the structural priming effects displayed by participants exposed to the extreme end of the fertility continuum. Participants who interacted with confederates with low conception risk (risk values<.01; 47 participants) matched the constructions produced by the confederate 62% of the time, which is significantly above the 50% level [*t*(46) = 5.71, *p*<.001]. Participants who interacted with confederates with higher conception risk (risk values>.05; 37 participants) matched the constructions produced by the confederate on 49.7% of the trials, a value that is not significantly different from 50% [*t*<1]. It appears that participants who interact with confederates with low conception risk show the traditional structural priming effect, and participants who interact with confederates with higher conception risk do not. In addition, our analysis shows that as participants' ratings of the confederates' flirtatiousness increased, the odds of them matching the constructions produced by the confederate increases (*p* = .03; see Figure 3). We discuss this effect in more detail in the General Discussion.

**Table 1:** Mixed Logit Regression Results from Experiment 1.

Predictor	Coefficient	SE	Wald Z	p-value	Odds Ratio
Intercept	.44	.54	.81	.42	1.55
Conception Risk	−.08	.03	−2.99	.003	.92
Flirtatiousness	.21	.10	2.19	.03	1.30

Our results are consistent with the second possibility considered in the introduction, namely that the detection of fertility cues would be associated with higher levels of non-conforming or creative behavior (such as not aligning one's linguistic choices with those of a conversation partner). Before discussing the implications of our results further, we report a second study that is aimed at strengthening the conclusion that male detection of fertility cues affects their linguistic choices. This study replicates Experiment 1, except that the participants were heterosexual females. The account sketched in the introduction of the paper suggests that males might mismatch the constructions produced by their female conversational partner as a means of displaying their fitness as a mate. If this is correct, we expect that heterosexual females will not show the same relationship between conception risk and linguistic choice as was demonstrated here.

## Experiment 2

### Method

#### Participants

The participants were 47 female undergraduate psychology students from Florida State University. Two participants who self-reported a homosexual orientation was excluded from the study (consistent with the practices used in Experiment 1).

#### Confederates and Experimenters

The confederates in this study were two undergraduate women who were not taking hormonal birth control. All experimenters were females taking hormonal birth control. The handling of the experimenters and confederates was identical to that in Experiment 1.

#### Materials

The materials used in this study were identical to those used in Experiment 1, except that to shorten the overall length of the experiment, the conformity measure from the previous study was not used in this experiment.

#### Procedure

The procedure was identical to that of Experiment 1.

#### Design and Analysis

The data were analyzed as in Experiment 1. We again employed a model-fitting approach that started with the effect of conception risk. Conception risk was not a significant predictor of match. None of the other variables were significant predictors of match. Thus, the final model reported below contains conception risk and the random slope across items associated with the risk variable.

### Results and Discussion

#### Preliminary Analyses

We conducted preliminary analyses to determine whether changes in conception risk were accompanied by other changes that might account for the effects of conception risk on structural priming. None of the participants' ratings of the confederates were significant predictors of conception risk (*p*'s>.13), and the combination of all of the variables did not significantly predict conception risk [*F*<1]. We also assessed whether there was a reliable structural priming effect in this study. Participants matched the syntactic construction produced by the confederates on 56% of the trials (*M* = .56, *SD* = .11), a figure that is significantly different than 50% [*t*(44) = 3.35, *p* = .002].

#### Main Analysis

Mixed logit analysis results for Experiment 2 are presented in Table 2. The critical result is that conception risk does not affect the odds of the participants matching the syntactic construction produced by the confederates (*p* = .93). Whereas changes in conception risk affected the extent to which heterosexual males matched the syntactic constructions produced by female confederates, heterosexual females display no such change in linguistic alignment as a function of conception risk. Caution is always in order when interpreting null results (particularly as the sample size of this study is somewhat smaller than the sample size of Experiment 1), but these data nonetheless suggest that the linguistic behavior of heterosexual males and females is affected in different ways by cues to female fertility.

**Table 2:** Mixed Logit Regression Results from Experiment 2.

Predictor	Coefficient	SE	Wald Z	p-value	Odds Ratio
Intercept	.32	.59	.54	.59	1.38
Conception Risk	−.004	.05	−.09	.93	.996

### General Discussion

Conception risk was inversely related to structural priming in heterosexual males: the higher the level of fertility in a female conversation partner, the lower the level of structural alignment men displayed. No such effect was observed in heterosexual females. The observed relationship between conception risk and structural priming is consistent with the second possibility considered in the introduction, namely that detection of fertility cues would be associated with higher levels of non-conforming or creative behavior (such as not aligning one's linguistic choices with those of a conversation partner). We follow the claims of Miller and Maner [20] in making the following proposal for how conception risk interacts with structural priming: 1) detection of fertility cues activates mating goals in men, 2) the activation of mating goals in turn leads to displays of fitness as a mate (such as creative or non-conforming behavior), and 3) non-conformity and creativity within our task manifested itself as the participants not aligning their syntactic choices with those of their partner. The data at hand do not allow us to determine whether the lack of alignment between males and the female confederates during periods of high fertility is best characterized as non-conformity or creativity. Whatever the case may turn out to be, both possibilities are consistent with the general claim that the reduction in alignment seen in Experiment 1 may be characterized as a display of fitness as a mate.

Although we are only beginning to scratch the surface with respect to understanding how conception risk affects structural priming, our data do provide some hints about the nature of the effect that is observed. The lack of an interaction between relationship status and conception risk (i.e., this predictor did not significantly add to model fit in Experiment 1, suggesting that the effect of conception risk was the same whether or not the male was in a committed relationship) suggests that the effect seen here reflects nonconscious, implicit changes in linguistic behavior. Miller and Maner [20], [22] demonstrated that relationship status affects men's responses to fertility cues when explicit behaviors are examined (e.g., providing ratings of the attractiveness of a woman), but not when implicit behaviors are examined (e.g., assessing the priming of concepts via a stem completion task). The idea that the effects of conception risk on structural priming reflect implicit, nonconscious behaviors on the part of the participant is consistent both with the theoretical position that structural priming reflects implicit learning in the language production system [26], [36], [37], and with claims that many sorts of behavioral and linguistic mimicry during interpersonal interaction occur on a nonconscious level [9], [20], [6], [38].

Participant ratings of the confederates' flirtatiousness were related to structural priming. Participants did not find the confederates to be especially flirtatious (mean rating = 1.95 out of 5), but those who did showed stronger structural priming. This result is consistent with the broad literature showing that conversational partners show affiliation by aligning their linguistic behavior [6]. Given that flirtatiousness and conception risk are both relevant to mating goals, it raises the question of why conception risk and flirtatiousness affected structural priming in opposite directions. We propose the following answer. When the participant perceives the confederate as flirtatious (i.e., he perceives interest on the part of the confederate), there is no need to signal fitness as a mate – the female has already signaled her interest. As such, the appropriate social strategy is to reciprocate the affiliation shown by the confederate. Within the context of our task, this can be accomplished by matching the structure of the utterances produced by the confederate. However, when the participant does not perceive the confederate as particularly flirtatious (as was likely the case for many of the participants in our study), the confederate has not signaled any particular interest in the participant. As such, when cues to fertility activate mating goals in the participant, the appropriate social strategy is to signal fitness as a mate in an effort to increase interest on the part of the confederate.

Although our reported effect of conception risk on structural priming is consistent with some elements of the literature on romantic relationships (particularly the idea that men may use non-conforming behavior to stand out to female conversation partners), the effect would appear to be at odds with a wide range of data suggesting that attraction to a conversational partner should lead to an increase in matching behavior [6], [12]. Indeed, at first blush the expectation that increases in fertility should lead to increases in alignment would appear to be the obvious prediction for our study. The contradiction between our data and previous work on alignment in conversation raises the possibility that there may be something unusual about our interaction setting that is driving the nature of the relationship between conception risk and structural priming. This concern is ameliorated to an extent by the finding that flirtatiousness leads to an increase in matching, as would be predicted on the “affiliation = alignment” view. Thus, our conversational task does reveal an expected social effect on alignment (as well as the traditional structural priming effect), but it appears that conception risk and the associated activation of mating goals may motivate speakers' behavior in a different way than the perception of flirtatiousness in the confederate.

Ireland et al. [12] note the paucity of research on linguistic behavior in relationships, and further note the importance of linguistic alignment (or a lack thereof) as a predictor of the promise and stability of a relationship. Our data add to this literature by suggesting that the role of linguistic behavior in the development of romantic relationships may not be as simple as the idea that people will align their linguistic behavior with that of attractive potential mates. Indeed, conversation partners may align their linguistic behavior (or not) based on a range of factors. If the potential mate has signaled interest in you, linguistic alignment may be a means of reciprocating that interest and developing a social bond. If the potential mate has not signaled an interest in you, non-alignment of linguistic choices may be a means of displaying one's fitness as a mate – and thereby capturing the potential mate's interest [21]. This proposal does not necessarily undermine the general claim that behavioral and linguistic alignment is an effective and commonly used means of building affiliation between individuals. Rather, it is intended to illustrate that linguistic behavior (aligning or non-aligning) can be driven by a range of social motivations, and that different social dynamics may affect both one's choice of behavior and the interpretation of that behavior. It is worth pointing out that even the act of aligning one's linguistic behavior with that of a conversation partner can serve multiple purposes – it can be affiliation-building in some cases, and affiliation-reducing in other cases (such as when the alignment is perceived as patronizing [6]).

Alignment is not an “all or nothing” variable. There are many different levels at which alignment can occur—both linguistically (e.g., sentence structure [8], lexical choices [39], and rate of speech [40]) and behaviorally (e.g., gestures [41], postures [42], and facial expressions [43]), and one can align on one level without aligning on other levels. The current study only examined one type of alignment—alignment of sentence structure. Therefore, we cannot determine whether men in our study diverged from fertile women on multiple levels or only on their choice of language structure. It may be the case that men in our study diverged from fertile women on sentence structure to accomplish certain goals (e.g., showing off their creativity or non-conformity to attract a mate) while aligning with them on other levels (e.g., rate of speech or vocal pitch) to accomplish other goals (e.g., affiliation). Previous research has not generally explored the extent to which alignment at one level corresponds to alignment at another level. The fact that fertility level affects the degree to which men align on linguistic choices differently than it affects the degree to which men align on behavior suggests that studying the relationship between different levels of interpersonal alignment may be a fruitful area for research.

We conclude with a broader point. For decades, social and cognitive approaches to language have had very little interaction (see [2] for a discussion). Our demonstration that a well-studied psycholinguistic phenomenon (structural priming) can be affected by social factors, combined with recent work on social aspects of language use [12], suggest that it may be profitable for researchers in both camps to pursue work at the intersection of cognitive and social approaches to language. It is our hope that findings such as these will spur interest in bridging these long-standing traditions of language research.
